# Three Synchronous Non-melanoma Skin Cancers of the Head and Neck: A Case Report and Literature Review

**DOI:** 10.7759/cureus.78020

**Published:** 2025-01-26

**Authors:** Eman Keshk, Adnene Moussa, Ammara Kashif, Abdullah Alsulaiman, Saud Alafghani, Nawal A Abdullah, Eyad S Alqurashi

**Affiliations:** 1 Histopathology Department, Ahmed Maher Teaching Hospital, Cairo, EGY; 2 Histopathology Unit, Laboratory Department, Al Hada Armed Forces Hospital, Taif, SAU; 3 Laboratory Department, Al Hada Armed Forces Hospital, Taif, SAU

**Keywords:** basal cell carcinoma, case report, histopathology, immunohistochemistry, squamous cell carcinoma, three tumors, ultraviolet light

## Abstract

Squamous cell carcinoma (SCC) and basal cell carcinoma (BCC) are the most common non-melanoma skin cancers, with exposure to ultraviolet (UV) light being the most important risk factor. These tumors usually affect older individuals with fair complexions. The co-occurrence of two or more non-melanoma skin cancers is a rare clinical event, the diagnosis of which rests on pathologic examination of the excised tumors. In this study, we report a case of the concurrent occurrence of three non-melanoma skin cancers on the head of a 90-year-old male patient with a history of long-term exposure to UV.

## Introduction

Squamous cell carcinoma (SCC) and basal cell carcinoma (BCC) are the most common non-melanoma skin cancers (NMSCs) of the head and neck [1]. Both typically occur in chronically sun-damaged skin of elderly people with fair skin tone [2]. Other risk factors include genetic syndromes (albinism, xeroderma pigmentosum, and nevoid BCC syndrome) and immunosuppression [3,4]. The synchronous presence of two different types of skin cancer is a rare event and usually occurs as collision tumors [5].

SCC is the second most common NMSC, with its incidence rising constantly in the past few years [6]. This increase is attributed to previously mentioned risk factors in addition to the strong relationship with previous skin burns and human papillomavirus (HPV) infection [7]; also, tumors can arise on top of predisposing dermatological conditions such as actinic keratosis, chronic wounds (Marjolin ulcer), porokeratosis, lichen sclerosus et atrophicus, hypertrophic or oral lichen planus, and discoid cutaneous lupus erythematosus [8].

SCC poses significant morbidity and mortality to patients, similar to melanoma and other aggressive malignancies, with lymph node metastasis being the most common metastatic route [8]. Early diagnosis and proper surgical and oncologic treatment are of paramount importance to improve the life expectancy of the patients [9]. Clinically, SCC presents as a scaly, erythematous, or hyperpigmented papule or plaque. Some cases may show ulceration, fungating mass, or pain [8].

Proper diagnosis requires pathological examination of a skin biopsy, with classic SCC showing nests of squamous epithelial cells arising from the epidermis and extending deep into the dermis. The neoplastic cells are generally large with abundant eosinophilic cytoplasm and large central vesicular nuclei with variably prominent nucleoli. Variable keratinization can be detected, with well-differentiated tumors showing keratin pearl formation [10,11].

SCC is graded as well, moderately, and poorly differentiated, depending on how easily the pathologist can recognize the characteristics of squamous epithelium (intracellular bridges and keratinization), pleomorphism, and mitotic activity [10,11]. Variants of SCC include spindle cell, clear cell, desmoplastic, follicular, keratoacanthoma-like, acantholytic, pseudovascular, metaplastic, inflammatory, basaloid, pigmented, infiltrative, pseudohyperplastic verrucous carcinoma, and adenosquamous carcinoma [11]. The differential diagnosis includes porokeratosis, actinic keratosis, seborrheic keratosis, BCC, melanoma, psoriasis, lichen planus, among others [12,13].

Classic treatment entails either Mohs micrographic surgery or simple excision with a 4-6 mm safety margin. The former is preferred in high-risk cases (lesions greater than 2 cm, high-risk histological features, recurrent lesions, and cosmetically sensitive locations such as the ears, lips, nose, and periocular regions), in addition to immunosuppression [14]. Prognostically, localized tumors generally have an excellent prognosis. Mortality rate for SCC is approximately 2% [15,16].

On the other hand, BCC is the most common NMSC [17], with the same risk factors for development as SCC. The tumor is more common in White people, while Black people are rarely affected [18]. Cutaneous development of BCC increases the risk of developing other skin cancers, including SCC and malignant melanoma [19].

BCC is a locally aggressive tumor with a low disease-associated mortality rate, and metastases are being exceptionally rare [20]. The tumor commonly presents clinically as a pearly pink or flesh-colored papule or nodule with arborizing and branching vessels. When ulceration occurs, it gives rise to the term rodent ulcer [21].

Histologically, the tumor has a lot of variants, with common and less common ones. Common variants include nodular and nodulocystic, infiltrative, morpheaform, keratotic, micronodular, adenoid, pigmented, basosquamous, superficial, ulcerative, and fibroepitheliomatous. Less common variants include clear cell, pleomorphic, signet ring cell, granular, infundibulocystic, metaplastic, keloidal, BCC with matricial differentiation, basomelanotic, BCC with thickened basement membrane, BCC with neuroid type nuclear palisading, and BCC with differentiation towards adnexal structures [11]. The nodular variant is the most common variant, presenting with circumscribed sheets and nodules of basaloid cells with epidermal attachment, peripheral palisading, stromal retraction, and possibly fibromyxoid change. There may be extensive cystic change. Pleomorphism is generally unremarkable [11].

Immunohistochemistry shows that the tumor cells are positive for cytokeratin AE1/AE3 (CK AE1/AE3), antihuman epithelial antigen BerEP-4, P63, CAM 5.2, cluster of differentiation 10 (CD10), 34BE12, and B-cell leukemia/lymphoma 2 protein (BCL-2) [22]. Histologic mimics of BCC include, dermatofibroma, trichoepithelioma, basaloid follicular hamartoma, SCC syringoma, microcystic adnexal carcinoma, Merkel cell carcinoma, among others. Differentiation relies on clinical features, thorough histological examination, and the application of appropriate ancillary immunohistochemical markers [23]. Treatment options include simple excision, Mohs surgery, topical 5-fluorouracil, radiation therapy, curettage, and electrodessication [24].

## Case presentation

A 90-year-old man presented to Al Hada Armed Forces Hospital with three skin tumors on his face. The first one was on the forehead, the second one on the nose, and the third one on the upper lip. The patient was a former military recruit with a long history of exposure to ultraviolet radiation. Grossly, the first tumor was excised by simple excision, with a skin ellipse and underlying subcutis. The skin showed a central irregular ulcer with a variegated base measuring 4 x 2.5 cm, 0.5 cm away from the nearest side margin. It was dissected in a cruciate manner. The second tumor was also removed by simple excision, with a skin ellipse and underlying subcutaneous tissue, and it showed an ulcerated elevated nodule measuring 3.5 x 2.5 x 1.5 cm, 0.2 cm away from the nearest margin. It was dissected in a cruciate manner. The same type of surgery was done for the third tumor, with a piece of skin showing a central pearly white nodule 0.7 x 0.7 cm, 0.2 cm away from the nearest margin, and it was similarly dissected in a cruciate manner.

**Figure 1:**
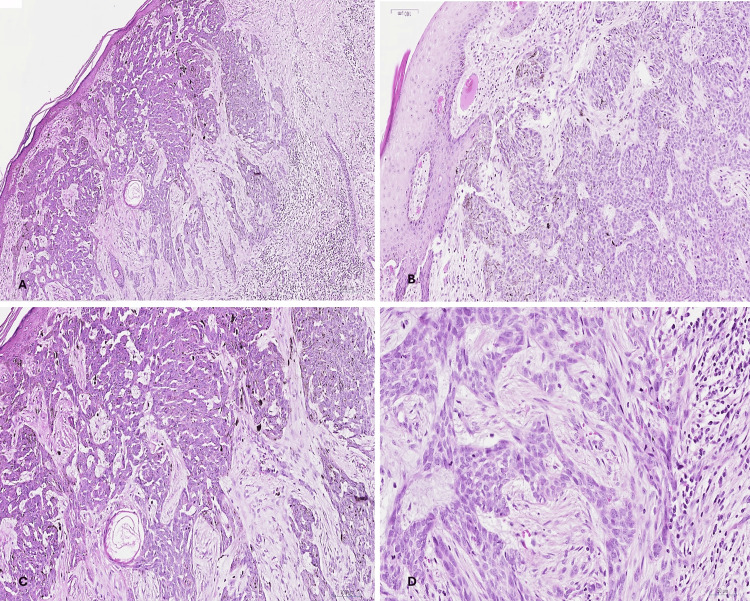
Forehead tumor (infiltrative BCC). (A): Low-power view showing the whole mount of the tumor, H&E ×40. (B, C) Intermediate magnification showing strands and trabeculae of intercommunicating tumor cells connected to the overlying epidermis, H&E ×100. (D) Higher magnification showing the basaloid nature of the tumor cells with mild pleomorphism. Note that peripheral palisading and stromal retraction are not marked in this subtype of BCC, H&E ×200. BCC: basal cell carcinoma; H&E: hematoxylin and eosin.

Histologic examination of the first (forehead) mass showed infiltrative BCC. The tumor was formed of infiltrating strands and interconnecting trabeculae of basaloid cells with mild peripheral palisading and stromal retraction, infiltrating among fibrocollagenous stroma. Tumor cells were connected to and ulcerating the overlying epidermis as well as focally reaching the subcutaneous fat, while all surgical margins were free of malignancy (Figure 1).

**Figure 2:**
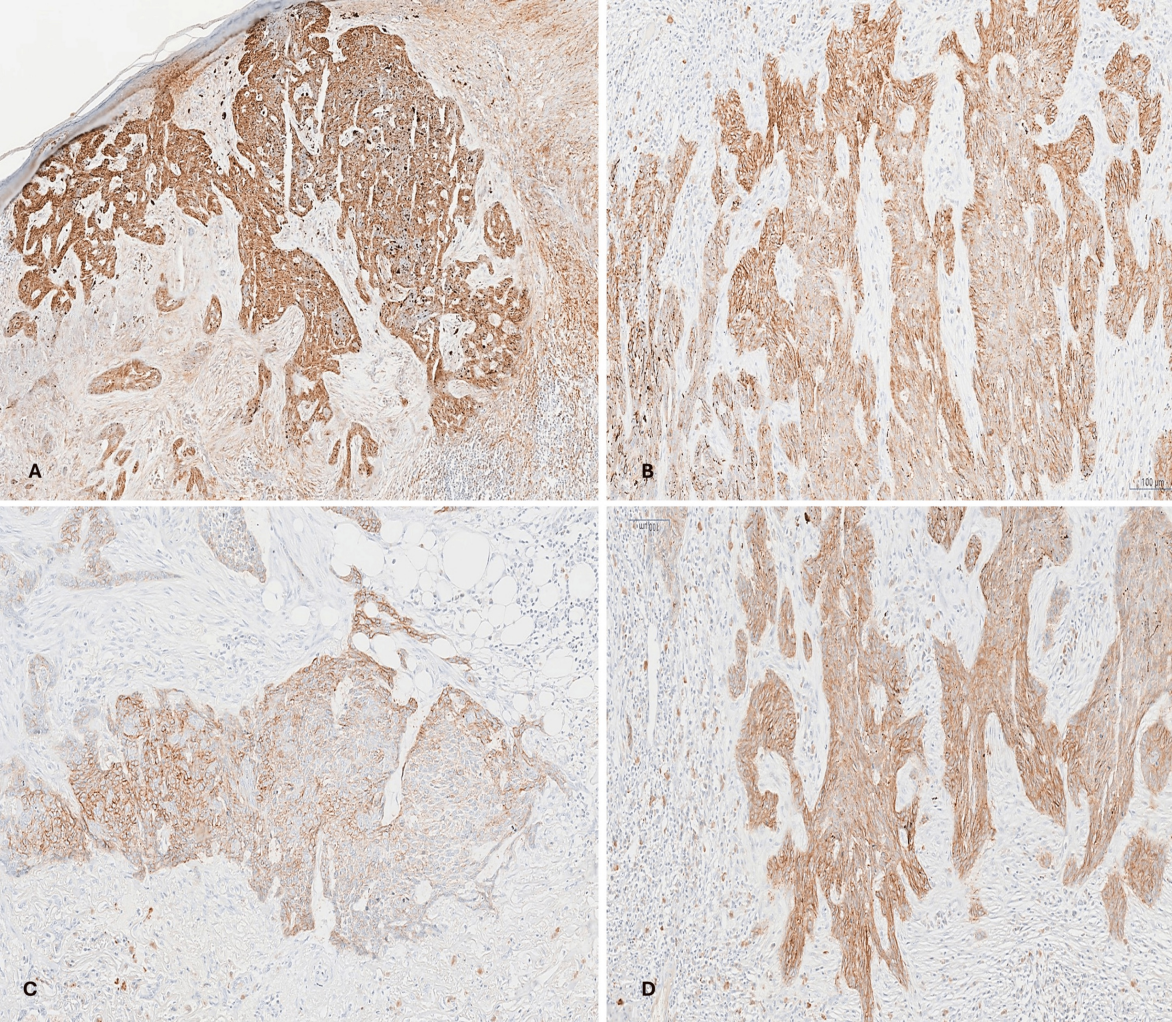
Forehead tumor (infiltrative BCC). (A, B) IHC showing positive membranous staining of the tumor cells for BerEP4, ×40 and ×100, respectively. (C, D) IHC showing positive membranous and cytoplasmic staining of the tumor cells for CD10, ×100. BCC: basal cell carcinoma; IHC: immunohistochemistry.

Immunohistochemical studies showed that the tumor was positive for the epithelial adhesion molecule (BerEP4) and cluster of differentiation 10 (CD10) (Figure 2).

**Figure 3:**
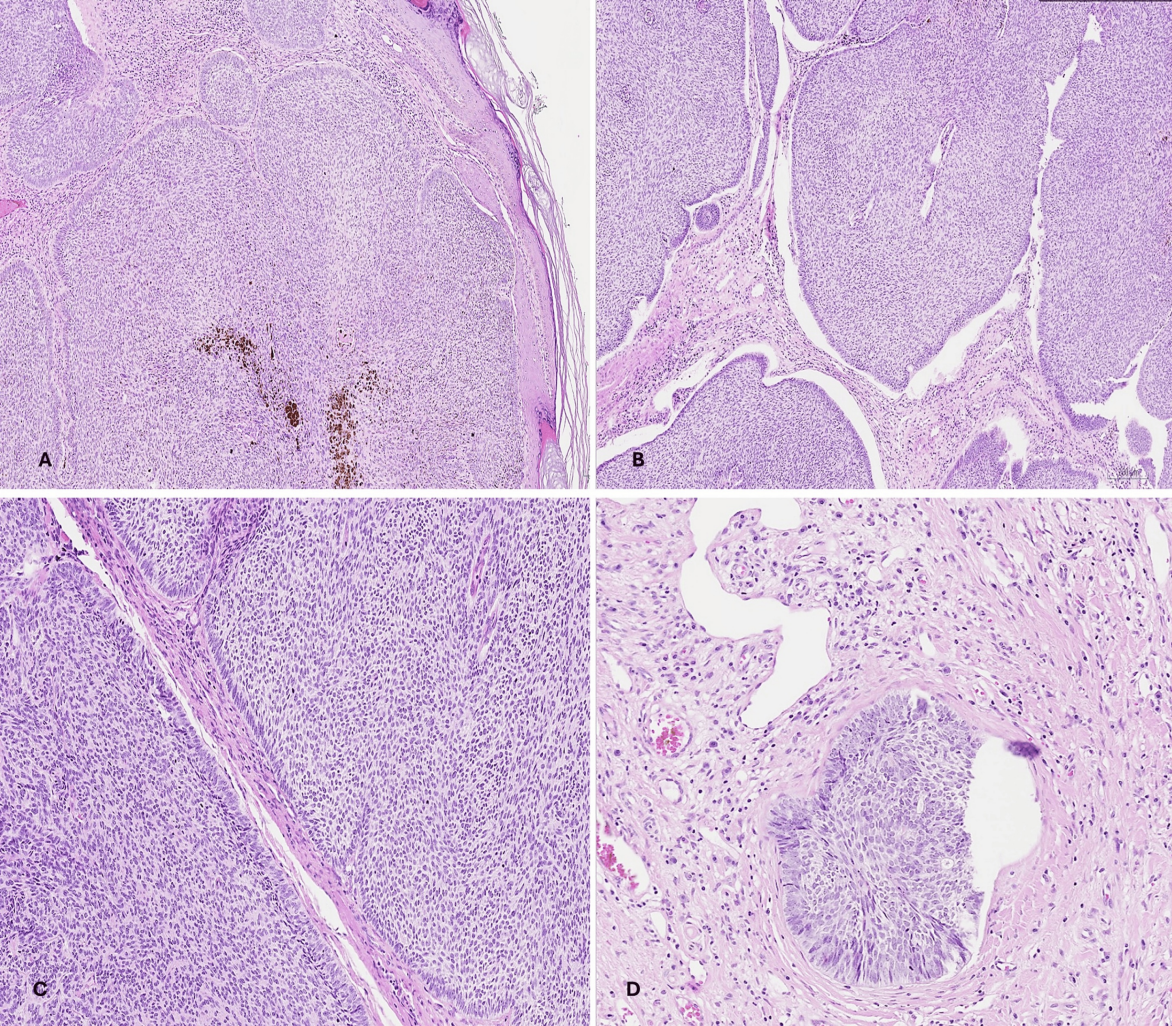
Nasal tumor (nodular BCC). (A, B) H&E showing circumscribed nodules of neoplastic basaloid cells, ×40 and ×100, respectively. (C) Tumor cells showing peripheral palisading and stromal retraction, H&E ×100. (D) Tumor nest was seen infiltrating blood vessel (lymphovascular invasion), H&E ×200. BCC: basal cell carcinoma; H&E: hematoxylin and eosin.

Histologic examination of the second (nasal) mass showed nodular BCC, formed of circumscribed nodules and sheets of neoplastic basaloid cells, with peripheral palisading and stromal retraction, with areas of identifiable lymphovascular invasion and infiltration of the deep dermis (Clark level IV). All surgical margins were free of malignancy (Figure 3).

**Figure 4:**
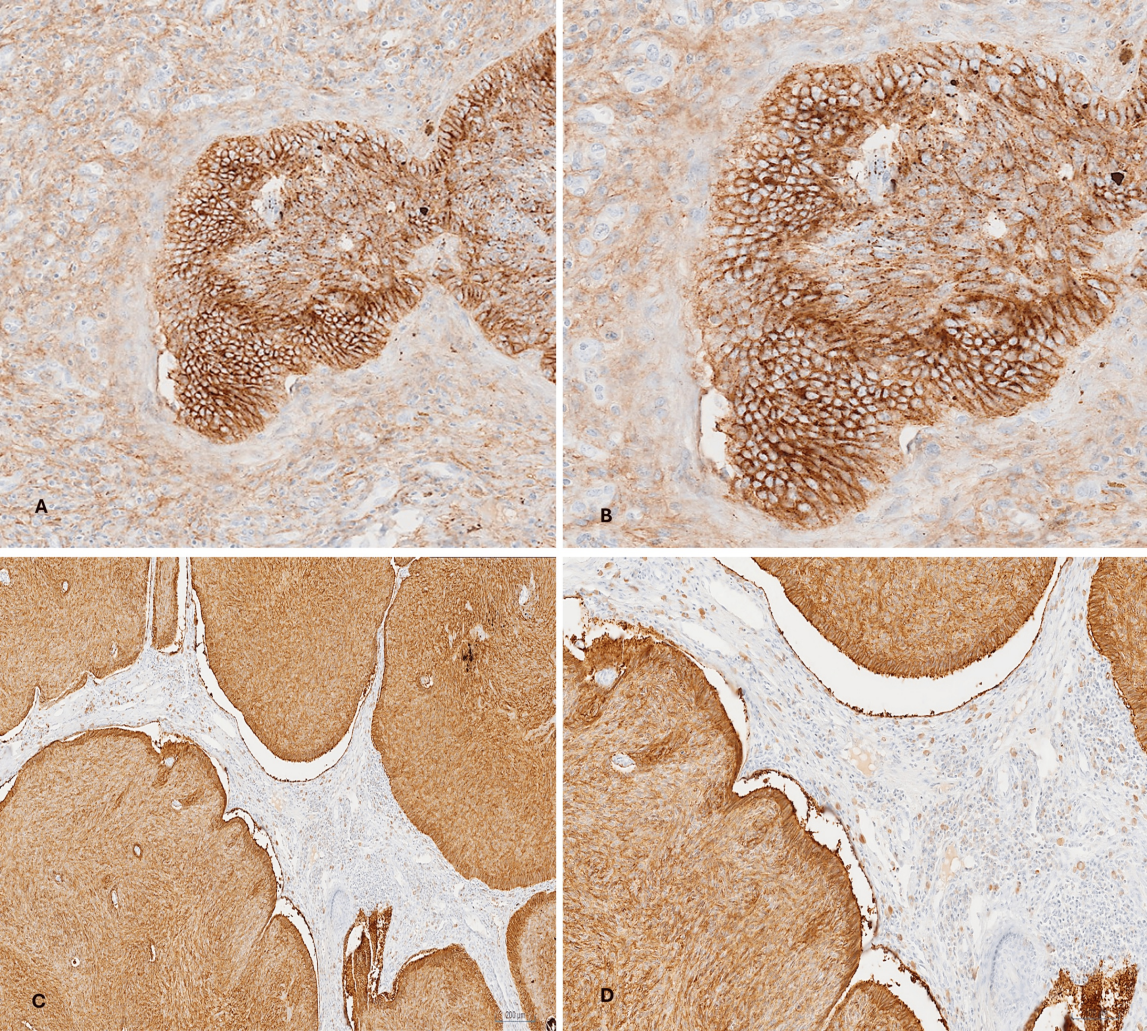
Nasal tumor (nodular BCC). (A, B) Positive membranous and cytoplasmic staining of the tumor cells for positive CD10. CD10 IHC, ×100 and ×200, respectively. (C, D) Tumor cell nodules showing positive staining for BerEp4. BerEP4 IHC, ×40 and ×100, respectively. BCC: basal cell carcinoma; IHC: immunohistochemistry.

IHC showed that the tumor was positive for BerEP4 and CD10 (Figure 4).

**Figure 5:**
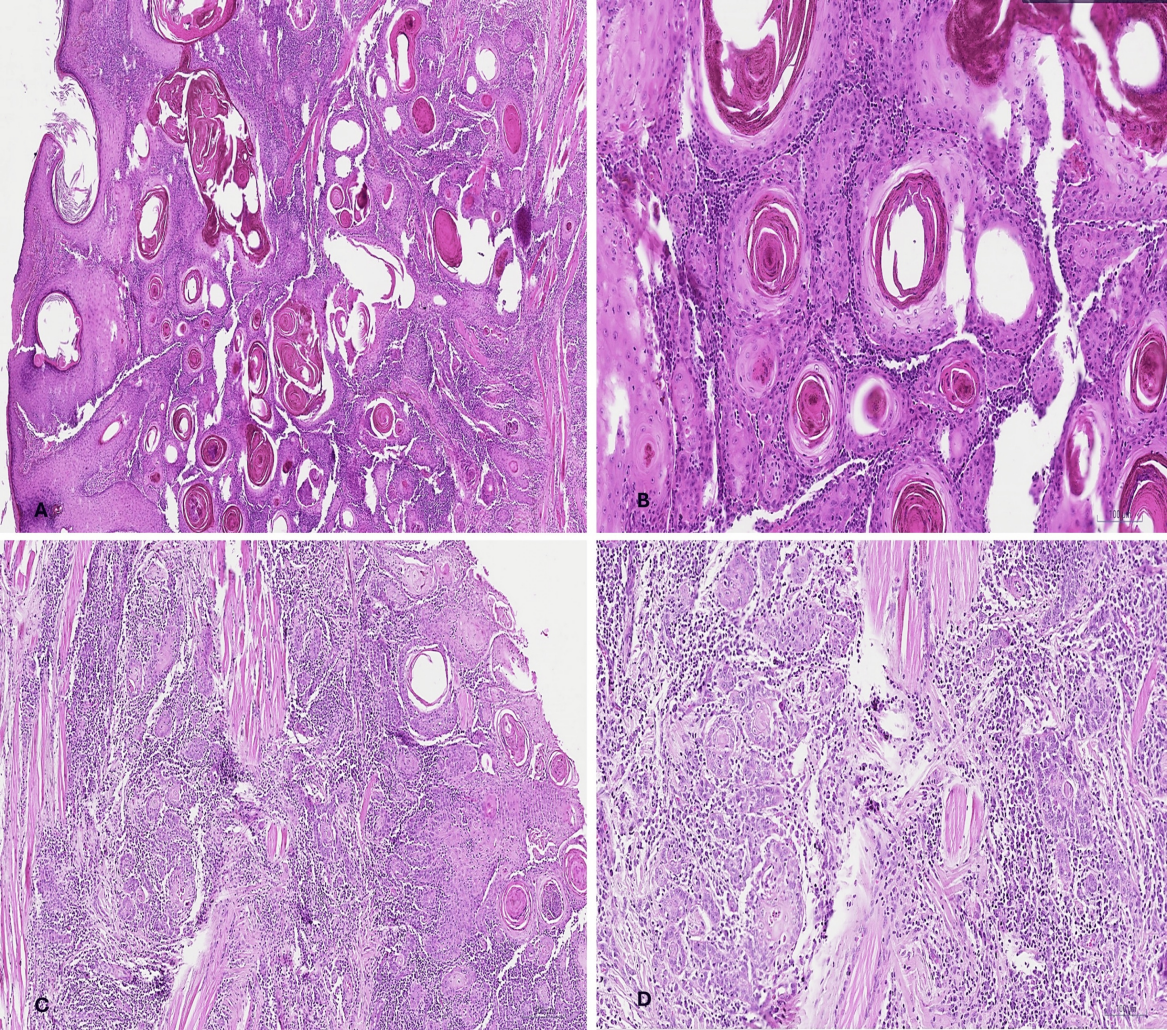
Upper lip tumor (well-differentiated SCC). (A, B) H&E-stained sections of the tumor showed well-differentiated SCC, with abundant keratinization, ×20 and ×100, respectively. (C, D) Nests of neoplastic squamous cells were seen infiltrating skeletal muscle bundles, H&E ×40 and ×100, respectively. SCC: squamous cell carcinoma; H&E: hematoxylin and eosin.

Histological examination of the third (upper lip) tumor showed invasive well-differentiated SCC, in which the tumor forms sheets and nodules of neoplastic squamous cells with mild pleomorphism and evident keratin pearl formation. The tumor was seen infiltrating facial skeletal muscles with areas of perineural invasion. All surgical margins were free of malignancy (Figure 5).

**Figure 6:**
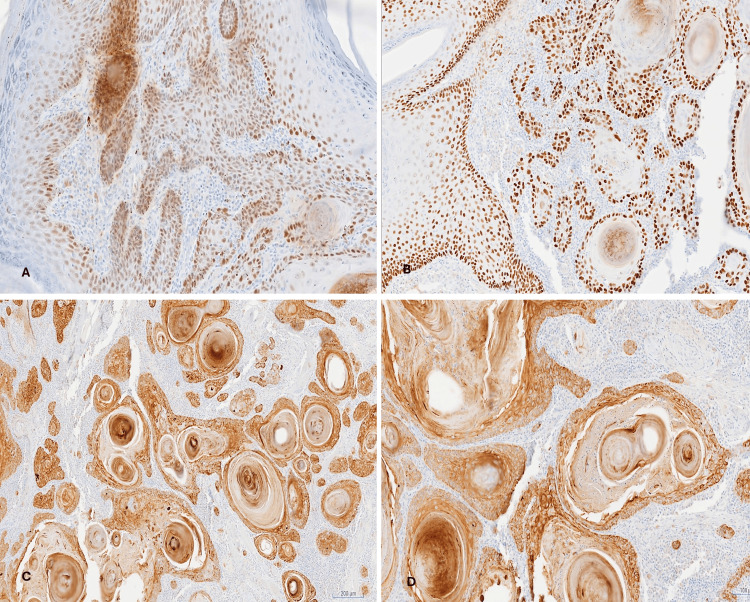
Upper lip tumor (well-differentiated SCC). (A) P63 showing positive nuclear staining of the tumor. P63, ×100. (B) P40 showing positive nuclear staining of the tumor cells. P40, ×100. (C, D) CK5/6 showing positive cytoplasmic staining of the tumor cells. CK5/6, ×40 and ×100, respectively. SCC: squamous cell carcinoma; CK5/6: cytokeratin 5/6.

Immunohistochemically, the tumor cells showed positive nuclear staining for P63 and P40, while cytokeratin 5/6 (CK5/6) showed positive cytoplasmic staining (Figure 6).

This case was diagnosed in October 2024. Follow-up of the patient shows favorable outcome, good cosmetic results, and no evidence of lymph node or distant metastasis.

## Discussion

Co-occurrence of multiple malignant skin tumors has been rarely reported in the past [25]. We reported a case of three synchronous NMSCs in the head and neck of a 90-year-old male patient, who was a previous military recruit: infiltrative BCC, nodular BCC, and SCC. Most studies have reported that the head and neck are the most common sites for these tumors, with the male gender more commonly affected than females [26].

Our reported variants of BCC are among the most common variants of BCC [11], while the SCC reported was a well-differentiated classic variant, which has been reported as the most common variant of SCC [11]. In our case, both carcinomas were completely resected with safe margins and plastic reconstruction. The standard treatment for cutaneous BCC and SCC is either Mohs surgery or simple excision with a 4-6 mm safety margin [27].

## Conclusions

In summary, this report documents a rare and unusual case of three synchronous NMSCs: infiltrative BCC, nodular BCC, and well-differentiated SCC, identified on the face of a 90-year-old male patient, who was a former military recruit. These tumors are more common in elderly male patients. Biopsies with histological assessment are the key to the correct diagnosis due to the variations in tumor morphology. A complete resection of all carcinomas with safe margins and plastic reconstruction was performed. Clinicians and pathologists need to be more cautious when working up such lesions.
